# A Rational Evaluation of the Syncope Patient: Optimizing the Emergency Department Visit

**DOI:** 10.3390/medicina57060514

**Published:** 2021-05-21

**Authors:** Tarek Hatoum, Robert S. Sheldon

**Affiliations:** Libin Cardiovascular Institute of Alberta, University of Calgary, Calgary, AB T2N 4Z6, Canada; tarek.hatoum1@ucalgary.ca

**Keywords:** syncope, emergency, risk stratification

## Abstract

Syncope accounts for up to 2% of emergency department visits and results in the hospitalization of 12–86% of patients. There is often a low diagnostic yield, with up to 50% of hospitalized patients being discharged with no clear diagnosis. We will outline a structured approach to the syncope patient in the emergency department, highlighting the evidence supporting the role of clinical judgement and the initial electrocardiogram (ECG) in making the preliminary diagnosis and in safely identifying the patients at low risk of short- and long-term adverse events or admitting the patient if likely to benefit from urgent intervention. Clinical decision tools and additional testing may aid in further stratifying patients and may guide disposition. While hospital admission does not seem to offer additional mortality benefit, the efficient utilization of outpatient testing may provide similar diagnostic yield, preventing unnecessary hospitalizations.

## 1. Introduction

Syncope is a transient loss of consciousness due to cerebral hypoperfusion. It is brief, reversible—with full recovery of neurologic baseline function—and does not require specific resuscitative measures. The incidence and prevalence of syncope vary among studies due to heterogeneity in the definitions and populations studied. A lifetime incidence of syncope is estimated to be at least 32–35% [1,2,3]. Women universally have a higher incidence of syncope than men, and the elderly fare worse compared to younger patients [4].

Syncope accounts for 0.6–1.7% of emergency department (ED) visits, and subsequent admission rates range from 12–86%, varying among countries and healthcare systems [5]. The economic impact of syncope care varies, as much of the cost is incurred for inpatient care. In countries with lower hospitalization rates, as in the case of Canada, the cost of syncope care is significantly less than countries with high hospitalization rates [6,7]. The most common cause of syncope is benign, with vasovagal syncope (VVS) and orthostatic hypotension (OH) accounting for up to two-thirds of the cases seeking ED care. Although estimates vary, syncope is ascribed to cardiac causes in about 7–10% of cases. Cardiac syncope is more common in older populations, and a significant portion remains unexplained following the ED visit [5]. In a multicenter prospective study following 5000 patients for one month after ED discharge [8], discharge diagnosis was presumed to be VVS in 53.3% of patients and cardiac syncope was diagnosed in 5.4%.

The mortality rate in patients presenting with syncope is estimated to be <2% at 10 days from the ED visit and 8.4% at one year (5.7–15.5%), with morbidity rates estimated to be 6.9% at 10 days and 25.2% at 2 years [9]. A Canadian study showed low short- and long-term mortality rates in patients discharged from the ED (30 days: 0.4%; one year: 3.0%), with mortality in hospitalized patients being three to four times higher at 30 days and one year [6]. The in-hospital mortality of syncope patients in Canada is estimated to be 0.9% [6]. Similarly, an Italian study found a much higher mortality of 14.7% in patients hospitalized with syncope compared to 1.8% mortality rate in syncope patients discharged from the ED [10]. Of note, almost half of admitted patients in this study were older than 65 years and had significant cardiac comorbidities. In a propensity score analysis of 8864 patients admitted from the ED within 48 h, Kaul et al. found that admitted patients were significantly older, lived in rural areas, were mostly males, and had lower income [11]. Neither short- nor long-term mortality was reduced in hospitalized patients compared to a matched cohort. Whether this is because sicker patients are hospitalized or whether hospitalization itself increases mortality is not well established.

Vasovagal syncope is the most common cause of syncope at all ages and is most common in the young. Other common and benign causes include initial orthostatic hypotension and classic orthostatic hypotension. Cardiac syncope, usually due to treatable arrhythmias or aortic stenosis, is more common in the elderly. While a fainting spell might appear to be a benign event, it must be scrutinized by the physician for potential treatable cardiac causes. As well, syncope can lead to body injuries of variable severities. About 15% of syncopal events lead to injuries, and 30% of syncope patients have been injured during a faint at some time [12,13]. Despite warning signs and prodromes, 33% of patients with VVS are injured during syncope at some point [14].

Syncope symptoms may be deemed severe enough to impair a patient’s lifestyle, career prospects, or psychological wellbeing [15]. However, the mortality risk varies considerably with underlying causes and co-morbidities and is not increased in VVS [16]. Classic OH carries a 1.5-fold mortality risk due to later major adverse cardiovascular events compared to patients with no OH, and the risk is more pronounced in patients under 65 years old [17]. Patients with syncope due to OH warrant referral for expert evaluation, mostly to address comorbidities. Syncope, regardless of symptoms, in the presence of structural or electrical heart disease warrants further assessment and possible urgent intervention. The ED physician carries the burden of accurately identifying those at higher risk of cardiac syncope who might benefit from urgent in-hospital evaluation and intervention while safely discharging the vast majority of the patients who are at low risk.

## 2. Value of History and Clinical Exam

The first step in syncope evaluation in the ED is confirming or excluding the occurrence of transient loss of consciousness and triaging the patient into one of four broad categories: traumatic or nontraumatic head injury (ischemic or hemorrhagic stroke), epileptic seizure disorders, psychogenic collapses, and true syncope [18]. Syncope is due to global cerebral hypoperfusion and is characterized by loss of muscle tone; it ends in collapse, brief unresponsiveness, transient amnesia, absence of focal neurologic signs, and subsequent full recovery.

Brief myoclonic convulsions are common and can lead to an initial assessment for seizure disorders. With up to 70% of syncope patients having convulsive activity, distinguishing convulsive syncope from seizures remains an important task, as misdiagnosing syncope for epilepsy is not uncommon [19]. A history of drug-refractory seizure disorder, the absence of a postictal state, convulsions lasting less than a minute, and myoclonic activity favor syncope rather than epilepsy. A point score developed by our group had 94% sensitivity and specificity to distinguish syncope from seizures [20].

A thorough history, including witness and first responder accounts when available, identification of risk factors for adverse outcomes, and a focused physical exam remain the key elements in identifying the high-risk patient [4,21,22]. Establishing a preliminary diagnosis after a standardized history, physical exam, and an electrocardiogram (ECG) can be achieved in almost two-thirds of patients with an 88% accuracy, thus avoiding unnecessary testing and hospitalization [23]. A diagnosis of VVS carries an excellent prognosis, while having an ED diagnosis of cardiac syncope predicts an unfavorable prognosis [8]. Having a history of heart disease, male gender, an age of more than 40 years, a lack of prodromes, and no more than two spells are major predictors of cardiac syncope [24], with heart disease being an independent risk factor for cardiac syncope [25]. Furthermore, in patients with known structural heart disease, a structured, evidence-based history can identify patients with ventricular tachycardia with 99% sensitivity, 68% specificity, and 96% negative predictive value [26]. The presence of nausea, diaphoresis, warmth, and dizziness before or after the faint are predictive of VVS, as is post-syncopal fatigue, prolonged prodrome, syncope while standing or sitting, and headaches [26,27]. In a meta-analysis of 11 syncope studies, a history of ischemic heart disease or heart failure, palpitations preceding syncope, syncope during exertion, and evidence of bleeding were strong predictors of adverse outcomes [28]. Table 1 summarizes high-risk features obtainable from patient history.

## 3. Value of the ECG

The ECG plays a crucial role, along with the history and physical examination, in excluding serious cardiac risk factors. Several studies and risk scores have identified “abnormal ECG” or “non-sinus rhythm” as predictors of adverse outcomes [29,30,31,32,33]. Non-sinus rhythm and any left bundle branch conduction abnormality carried threefold odds ratios of significant cardiac outcomes in patients with syncope or near syncope who were more than 35 years old [34]. The heterogeneity and inclusive non-specificity of abnormal ECG definitions in almost all early studies prompted Ottawa investigators to identify more specific ECG risk factors. The Ottawa Electrographic Criteria identified the presence on ECG of high-grade atrioventricular block, any bifascicular block, non-sinus rhythm, new ischemic changes, left axis deviation, or ED monitoring abnormalities as strongly predictive of 30-day serious cardiac outcomes in adults [35]. In a prospective study, this group identified non-sinus rhythm and prolonged QTc as independent predictors of 30-day arrhythmia or death [36]. Although a large proportion (30–65%) of adult patients with syncope have an abnormal ECG, only the presence of atrial fibrillation, intraventricular conduction delay, left ventricular hypertrophy, and pacemaker rhythm were independently associated with one-year mortality [37]. A recent prospective multicenter study found non-sinus rhythm, multiple premature ventricular contractions, short PR interval, first-degree atrioventricular block, complete left bundle branch block, and ischemic Q/ST/T-segment abnormalities to be associated with a two- to threefold increase in 30-day serious cardiac arrhythmias in syncope patients older than 60 years, with similar sensitivity to other findings of abnormal ECG but with slightly better specificity [38]. Table 2 highlights the high-risk ECG features in young and older adults. Prolonged QTc in an older population (>60 years old) in sinus rhythm and no conduction abnormalities was not an independent risk marker for 30-day serious adverse outcomes [39]. However, in smaller retrospective studies with more than 30 months mean follow-up, QTc > 450 ms in men and >460 ms in women carried a 2.2-fold hazard ratio for long-term mortality [40], and QTc > 500 ms in patients older than 65 years was associated with a 3.5-fold hazard ratio for mortality [41]. Early repolarization patterns did not seem to increase risk in this population or in patients referred for tilt testing and followed up for two years [42].

In a recent prospective multicenter international study, the BASEL-IX investigators sought to integrate several ECG criteria to develop an ECG-based risk tool to identify cardiac syncope in patients older than 40 years presenting to the ED [44]. The model identified seven independent predictors of cardiac syncope that were used to build the “Basel ECG Risk Calculator for Cardiac Syncope” (ALERT-CS); these predictors are shown in Table 3. Based on a predefined 99% sensitivity and 95% specificity, a predicted probability of 5.5% was set as a rapid rule-out and a derived probability of 37.5% for rule-in cardiac syncope. The model showed a high diagnostic accuracy, with AUC of 0.8—better than the EGSYS and SFSR. Using the ECG model alone identified 11% of the high-risk and 8% of the low-risk patients. The tool also increased the accuracy of clinical judgement and biomarkers significantly. Based on this prediction tool, 30-day major adverse cardiac events were 37.5 times higher in the rule-in cohort compared to the rule-out group. The results of the BASEL-IX were prospectively validated with the Syncope Risk Stratification in Older Adults cohort, showing similar accuracy to the derivation cohort [38,39].

## 4. Risk Stratification

Several risk scores using ECG and history parameters have been developed to refine patient stratification, estimate prognosis, and guide decisions for admission or urgent specialist referral, including the Martin-Kapoor score [33], the San Francesco Syncope Rule (SFSR) [29,45], the OESIL score (Osservatorio Epidemiologico sulla Sincopenel Lazio) [31], and the EGSYS score (Evaluation of Guidelines in Syncope Study) [32]. However, the SFRS performed poorly in validation studies due to high miss rates [46,47,48]. The Basel ECG calculator showed better accuracy compared to EGSYS and SFSR [44]. When compared to the simple and commonly used CHADS2 score (congestive heart failure, hypertension, age > 75 years, diabetes (all 1 point each); previous stroke (2 points)), the CHADS2 score had equal or better accuracy in predicting one-year mortality and major adverse cardiac events (MACE) [49]. On the other hand, early clinical judgement performed better than those syncope risk rules for diagnosing cardiac syncope. In another study, clinical judgement had similar sensitivity but better specificity for recognizing patients at high risk for short term adverse events [50], although the scores predicted fatal outcomes to a better extent. A meta-analysis utilizing individual patient data failed to find additive value of the OESIL, EGSYS, and SFSR beyond clinical judgement to predict serious adverse outcomes in the ED or at 10 and 30 days [51]. Solbiati et al. used attribute matching—a tool that allows for personalized risk prediction by computer generated modeling—in an effort to refine 10-day risk prediction of serious adverse events, as compared to clinical judgment and a regression model [52]. The matching cohort included 3388 patients from five previous prospective trials. Attribute matching was found inferior to regression models and required a much larger cohort to match to all the variables. Due to inconsistent validity of these rules, both American and European guidelines give prediction tools a class IIb recommendation [21,22].

**Figure 1:**
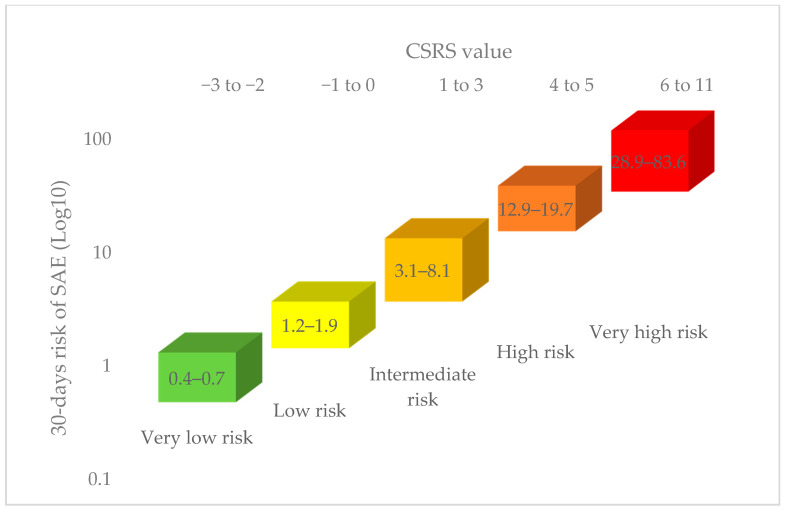
Risk prediction at 30 days from emergency evaluation based on CSRS value and risk category [43]. CSRS: Canadian Syncope Risk Score, SAE: serious adverse events.

The Canadian Syncope Risk Score (CSRS) was developed to overcome limitations observed in the earlier clinical decision tools [43]. The study prospectively evaluated 4030 adult syncope patients presenting to six Canadian emergency departments. The point score model ranges from −3 to 11, and includes clinical data, investigations, and presumptive ED diagnosis, as shown in Table 4. The model had a 99.2% sensitivity for predicting 30-day serious events for a score of −2 or higher. Figure 1 shows the estimated 30-day risk of serious adverse events (SAE) according to CSRS value and risk category. Following the publication of recent syncope guidelines, the CSRS was validated in nine Canadian centers [53]. The observed risk of 30-day serious events was 0.3% in the very-low-risk cohort compared to 51% in the very-high-risk group. The model had a 97.8% sensitivity and 44.3% specificity at a score of −1 or higher. An Australian single center validation study in 283 patients, however, demonstrated a lower sensitivity (71.4%) of a CSRS −1 [54]; similar sensitivity was maintained at a CSRS threshold of 1, with improved specificity (72.8%), and maintained a negative predictive value of 99%.

In a recent study comparing cardiac biomarkers to available risk scores, the CSRS performed better than not only cardiac biomarkers at predicting death and adverse outcomes, but also cardiac biomarkers combined with older risk scores [55].

## 5. Other Diagnostic Tests in The Emergency Department

**Figure 2:**
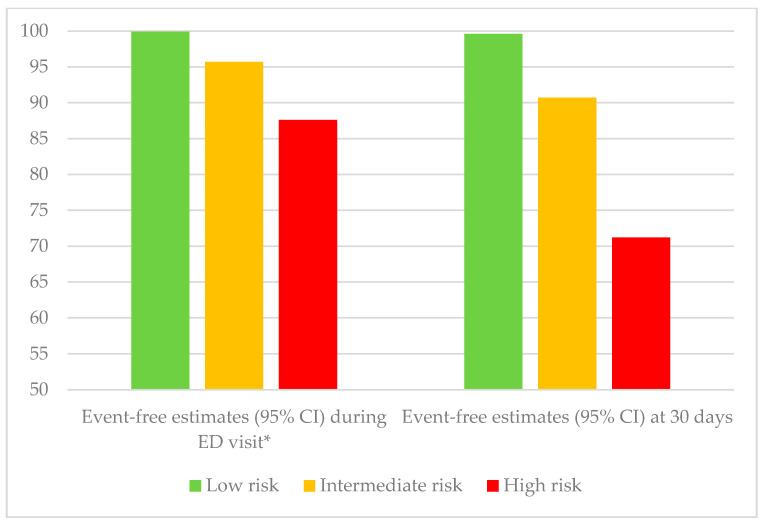
Serious event- and arrhythmia-free estimates based on Canadian Syncope Risk Score (CSRS) classification from the time of emergency department (ED) presentation. * Within 2 h for low-risk and 6 h for intermediate- and high-risk patients and within 30 days [56].

Continuous cardiac rhythm monitoring remains an important complementary tool to 12-lead resting ECG while the patient is observed in the ED. The duration of ED monitoring is controversial. In low-risk patients identified by the CSRS, a 2 h monitoring period appears extremely safe in excluding serious arrhythmic events [56]. However, for medium- and high-risk patients, despite identifying almost half the arrhythmic events within 6 h, a 5–18% residual risk remained, with 92% of arrhythmic events being identified within 15 days. Figure 2 summarizes the event-free estimates during the ED monitoring period and at 30 days.

Cardiac biomarkers were thought to improve diagnostic yield in identifying cardiac causes of syncope, but biomarker testing should be driven by relevant history and ECG findings. A patient-level meta-analysis of 4246 adult patients from 11 studies was performed and found only modest ability of brain natriuretic peptide (BNP) and high-sensitivity troponin (Hs-cTn) assays to identify the cardiac causes of syncope. It failed to predict patients at risk of major cardiac events [57]. A recent prospective trial compared the utility of BNP, pro-BNP, and Hs-Troponins in patients older than 45 years to the EGSYS risk score [55]. Biomarkers showed superior diagnostic accuracy for cardiac syncope, with an AUC of 0.77–0.78, and ability to rule in or out almost 30% of patients. However, although prognostic value was superior to the Risk Stratification of Syncope in the Emergency Department (ROSE), OESIL, and SFSR scores, its prognostic accuracy was inferior to the CSRS. NT-Pro BNP was significantly elevated in syncope patients older than 16 years and predicted serious adverse events occurring within 30 days of syncope presentation. However, it had no incremental prognostic value above the CSRS [58].

Avoiding unnecessary testing in the ED preserves healthcare resources and reduces ED visit times. In a study aimed at predicting significant echocardiographic findings, the ROMEO (Risk of Major Echocardiographic findings in Older syncope patients) criteria were developed [59]. The investigators enrolled 915 patients older than 60 years who presented with syncope and had an echocardiography performed. Regression analysis identified five variables (history of congestive heart failure or coronary artery disease, abnormal ECG, elevated Hs-cTn or NT-pro BNP), with 99.5% sensitivity to exclude serious echocardiographic findings; however, this tool has yet to be validated.

## 6. The Role of Hospitalization and Outpatient Referral

**Figure 3:**
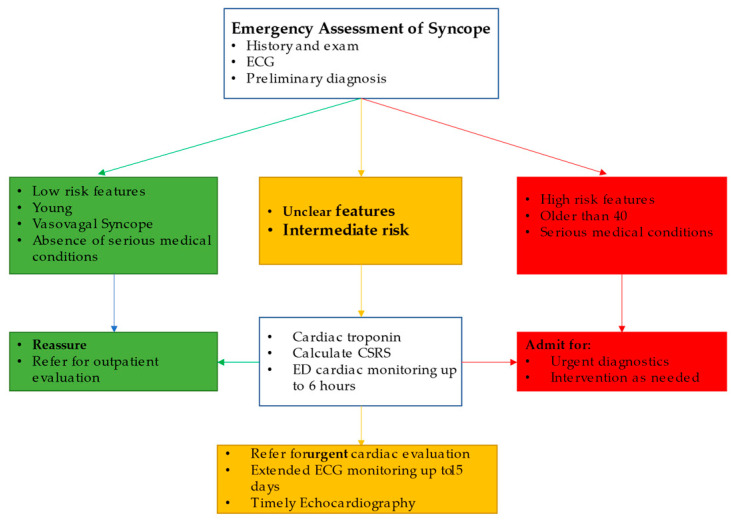
The authors recommended approach to syncope patients in the ED based on history and physical exam, ECG, ED diagnosis, and CSRS.

Discharging low-risk patients from the ED and admitting high-risk patients for further management is a reasonable strategy, but hospital admission remains controversial for patients with syncope of unclear etiology or moderate risk. The US guidelines recommend hospital admission based on the critical nature of ED diagnosis [22], while the European guidelines recommend admission on the basis of high-risk features upon evaluation [21]. Probst et al. found no significant difference after 30 days in serious events in syncope patients more than 60 years old with no serious ED diagnosis in American patients who are admitted to hospital when compared to the discharged group (propensity analysis) [60]. In a province-wide analysis from Alberta, Canada, hospitalized syncope patients had higher 30-day (3.6% vs. 0.3%) and one-year (14.3% vs. 3.0%) mortality rates when compared to discharged patients [11]. Upon discharge, 43% of the patients were discharged with a primary diagnosis of syncope, while the rest were discharged with an alternate diagnosis. The mortality seemed to be related to underlying comorbidities rather than the index syncope, and hospital admission did not appear to reduce mortality in this group. These findings confirm the lack of one-year mortality benefit from hospitalization and the impact of comorbidities on outcomes observed in a previous study [10]. In a Canada-wide study, 743 patients admitted to hospital were matched to 658 patients discharged from the ED [61]. The odds ratio of detecting an adverse outcome in hospitalized patients during admission was fivefold higher than in those discharged and over the next 30 days. This was mainly driven by detecting non-fatal arrhythmias and non-arrhythmic serious events, and no significant mortality difference was found. This difference between groups was more pronounced in patients older than 60 years, with an odds ratio of 7.7. The detection rates were found to be higher in patients in the CSRS intermediate or higher risk category. The contrast in results from the American and Canadian studies cited above may be explained by higher threshold for admission, where only 9% of the ED patients in the Canadian study were admitted compared to 75% from the American study. Thus, hospital admission in most patients did not seem to offer meaningful intervention that justifies hospitalization, with its associated costs and hospital-related adverse events [62]. A reasonable alternative to hospitalization can be a timely outpatient referral and appropriate directed testing. Cook et al. showed that in an ED syncope cohort that was directly discharged from the ED, 22% received referrals for outpatient cardiac testing; however, only 55% of the high-risk CSRS (>3) profile patients and 40% of the non-high-risk patients actually received cardiac testing [63]. Of the high-risk patients who did not get cardiac testing, 10% suffered out-of-hospital arrhythmias over a 30-day period. Cardiac rhythm monitoring should be efficiently utilized in this population, as 92% of arrhythmic events occurred within 15 days of the index syncope [56]. Figure 3 outlines our recommended approach to the syncope patient in the ED.

## 7. Conclusions

Clinical judgement based on proper history, ECG findings, and the most likely ED diagnosis remains the primary tool to approach the syncope patient and guide further management. Low-risk patients can safely be discharged with proper follow up when needed, while the high-risk patients with clear need for intervention benefit from further in-hospital management. When in doubt, the clinician may benefit from implementing other validated clinical decision tools and additional ED testing and monitoring; specifically, the CSRS appears to perform better than older scores in classifying patient risk. The Basel IX ECG ALERT-CS tool to identify high- and low-risk groups appears promising. Hospitalization, however, does not seem to offer meaningful diagnostic benefits, and unnecessary hospitalizations can be avoided by efficient outpatient cardiac referral and testing.

## Figures and Tables

**Table 1:** High-risk historical features for syncope [24,28].

Major High-Risk Clinical Features
Male sex
Brief or no prodromes *
Age > 40 years
Palpitations preceding syncope *
Age in 10-year increments
Syncope during effort *
Structural heart disease *
Syncope while supine *
Clinical evidence of bleeding
No more than 2 spells *

* Evidence from index presentation or past history.

**Table 2:** High-risk ECG features predictive of 30-day adverse outcomes [38,43].

Patients Older Than 16 Years	Patients Older Than 60 Years
QRS duration > 130 ms	Non sinus rhythm
QTc interval > 480 ms	Multiple PVCs
QRS axis < −30° or >100°	Short PR interval
	Acute or chronic ischemic changes
	Left bundle branch block
	First degree atrioventricular block

**Table 3:** The ECG variable predictors of cardiac syncope in patients older than 40 years in the ALERT-CS ^†^ calculator [44].

ALERT-CS ECG Variables
Heart rate *
Non-sinus rhythm
Corrected QT interval *
Ventricular ectopy
ST segment depression
Bundle branch block
Atrio-ventricular block

* Continuous variables. ^†^ BAseL ECG Risk CalculaTor for Cardiac Syncope (ALERT-CS)

**Table 4:** The Canadian Syncope Risk Score [43].

	Risk Factors	Points
Clinical Evaluation	Predisposition to VVS	−1
	CVD	1
	SBP < 90 or >180 mmHg	2
Investigations	Elevated troponin	2
	QRS axis < −30° or >100°	1
	QRS duration > 130 ms	1
	QTc interval > 480 ms	2
Clinical Diagnosis	ED diagnosis of VVS	−2
	ED diagnosis of cardiac syncope	2

Note: The Canadian Syncope risk score is used to identify patients with syncope at risk of SAE within 30 days after disposition from the emergency department. The score is obtained by adding the points of each risk factor. BP: systolic blood pressure, VVS: vasovagal syncope, ED: emergency department, CVD: history of cardiovascular disease.
